# Dog ownership, glycaemic control and all-cause death in patients with newly diagnosed type 2 diabetes: a national cohort study

**DOI:** 10.3389/fpubh.2023.1265645

**Published:** 2023-12-15

**Authors:** Karin Rådholm, Peder af Geijerstam, Mark Woodward, John Chalmers, Margareta Hellgren, Stefan Jansson, Olov Rolandsson

**Affiliations:** ^1^Department of Health, Medicine and Caring Sciences, Linköping University, Linköping, Sweden; ^2^The George Institute for Global Health, University of New South Wales, Sydney, NSW, Australia; ^3^School of Public Health, Imperial College London, London, United Kingdom; ^4^Department of Public Health and Community Medicine/Primary Health Care, The Sahlgrenska Academy at the University of Gothenburg, Gothenburg, Sweden; ^5^The Skaraborg Institute, Skövde, Sweden; ^6^School of Medical Sciences, University Health Care Research Centre, Örebro University, Örebro, Sweden; ^7^Department of Public Health and Caring Sciences, Uppsala University, Uppsala, Sweden; ^8^Department of Public Health and Clinical Medicine, Family Medicine, Umeå University, Umeå, Sweden

**Keywords:** diabetes mellitus type 2, dogs, glycaemic control, lifestyle, epidemiology, mortality

## Abstract

**Aims:**

To evaluate whether dog ownership from the time of type 2 diabetes diagnosis improved glycaemic control, increased achievement of major guideline treatment goals or reduced the risk of all-cause death.

**Methods:**

Patients diagnosed with type 2 diabetes were followed by linkage of four Swedish national registers covering diabetes, dog ownership, socioeconomics, and mortality. Linear regression was used to estimate the mean yearly change in glycated haemoglobin (HbA1c). Cox survival analysis and logistic regression were used to analyse associations between dog ownership and all-cause death and achievement of treatment goals, respectively.

**Results:**

Of 218,345 individuals included, 8,352 (3.8%) were dog-owners. Median follow-up was 5.2 years. Dog-owners had worse yearly change in HbA1c, and were less likely to reach HbA1c, low-density lipoprotein (LDL), and systolic blood pressure (SBP) treatment goals than non-dog-owners (adjusted odds ratios [95% CI] of 0.93 [0.88–0.97], 0.91 [0.86–0.95], and 0.95 [0.90–1.00], respectively). There was no difference in the risk of all-cause death (adjusted hazard ratio [95% CI] 0.92 [0.81–1.04], dog owners versus not).

**Conclusion:**

Owning a dog when diagnosed with diabetes did not lead to better achievement of treatment goals or reduced mortality, but was in fact associated with a smaller reduction in HbA1c and reduced likelihood of achieving treatment goals.

## 1 Introduction

Diabetes is a prevalent chronic disease with approximately 537 million diagnosed adults over 18 years of age worldwide in 2021 (1). The prevalence has increased rapidly over the last 20 years, largely due to the global trends of increasing overweight/obesity and physical inactivity (1). In Sweden, close to 500,000 persons (5% of the population) are diagnosed with type 2 diabetes (2). People with type 2 diabetes are at a greater risk of cardiovascular disease (CVD) and cardiovascular death compared to those without (3). To reduce complication rates and increase quality of life, the treatment cornerstones are physical activity, healthy diet, avoidance of tobacco use and of excessive use of alcohol, as well as medication for hyperglycaemia, hypertension, and dyslipidaemia (4). However, there are few patients with type 2 diabetes who achieve the recommended goal of 150 min of physical activity per week (5). Barriers for being physically active are lack of time, unsafe living environments, and thus fear of exercising outdoors, and lack of companionship for exercise (6).

Dog ownership has previously been found to lower the mortality risk in the general Swedish population (7). Adults who own a dog were more physically active than those who did not own a dog (8, 9) whilst dog owners walked approximately 25% more steps on a daily basis compared to non-dog owners (10). Getting a dog also increased leisure walking (11). Apart from the health benefits from being more physically active as a dog owner, there is also evidence of an association between owning a dog and reduced psychological stress and increased mental wellbeing (12, 13). Psychological stress might have a role in the development of type 2 diabetes in initially healthy populations and could affect outcomes in people with an existing diagnosis (14). However, not all studies have shown health benefits in those owning a dog. For example, dog ownership has been associated with an increased risk of initiation of treatment for hypertension and hyperlipidaemia (15). Thus, whether owning a dog is beneficial in terms of the owner’s cardiovascular health or type 2 diabetes disease progression, is currently unclear. The aim of our study was to evaluate whether dog ownership at the time of type 2 diabetes diagnosis improved glycaemic control, or affected all-cause death or achievement of guideline treatment goals for systolic blood pressure (SBP) and low-density lipoprotein (LDL).

## 2 Materials and methods

Patients with type 2 diabetes owning a dog, or living with a family member that owned a dog 1 year before- to 5 years after their type 2 diabetes diagnosis (henceforth referred to as dog owners), were compared to patients with type 2 diabetes that were not dog owners (as defined above) in a Swedish nationwide cohort followed in an individual-level study by linkage of four nationwide registers: (1) The Swedish National Diabetes Register (NDR) that provided data on type 2 diabetes diagnosis and all clinical diabetes-related data (HbA1c, blood pressure, blood lipids, antihypertensive treatment, lipid-lowering treatment, level of physical activity, smoking status, and body mass index [BMI]). (2) Statistics Sweden provided data on socioeconomic factors, including education, income, area of living, age, and sex, as well as possible spouse ownership of a dog for non-dog owning patients with type 2 diabetes. In case of death during follow up, Statistics Sweden also provided the date and cause of death. Data for socioeconomics for participants diagnosed between 2011 and 2016 are from 2010. (3) The dog register of the Swedish Board of Agriculture, as well as (4) the Swedish kennel club register, which provided data on dog ownership and dog breed. The dog register of the Swedish Board of Agriculture is a national, mandatory register for dog owners.

Patients diagnosed with type 2 diabetes 2006–2016, and with at least two HbA1c measurements, 1 year apart or more, were included. The primary outcome was glycaemic control evaluated by yearly change of HbA1c, which is the recommended marker of glycaemic control, and for which reduction is associated with a decrease in cardiovascular complications (16). Secondary outcomes were all-cause death (with individuals followed from inclusion until December 31st 2016) and reaching guideline HbA1c, SBP, and LDL treatment goals defined as HbA1c of below 52 mmol/mol (6.9%), SBP of less than 140 mmHg and LDL of less than 2.5 mmol/L (17). Self-reported lifestyle changes in physical activity and smoking status during follow up were evaluated as well.

### 2.1 Ethics statement

The study was approved by the regional ethical review board in Umeå (2016/44-31).

### 2.2 Patient and public involvement statement

It was not appropriate or possible to involve patients or the public in the design, or conduct, or reporting, or dissemination plans of our research.

### 2.3 Statistical methods

Categorical variables were summarized as the number of patients, with corresponding percentage, and differences between groups were tested using chi square tests. Continuous variables were summarized as mean (SD) or, for those with a skewed distribution [evaluated by an Anderson-Darling test (18)], median (interquartile interval), and differences between groups were tested using a one-way ANOVA for those with approximate normal distributions and a Mann–Whitney U test for those with skewed distributions. Prior to analysis, all registered creatinine and eGFR values above 150 (mL/min/1.73 m^2^) were removed to avoid bias, since these were assumed to be due to manual error. Missing data were handled by listwise deletion.

A linear regression model was used to estimate the primary outcome, mean yearly change of HbA1c, unadjusted and adjusted for sex, age at diabetes diagnosis, current smoking status, first HbA1c value, time to first recorded HbA1c from diabetes diagnosis, diabetes treatment, antihypertensive treatment, SBP, BMI, LDL, lipid-lowering treatment, income quarter, education, and rural/urban area of living. Secondary outcomes – reaching treatment goals (HbA1c <52 mmol/mol [<6.9%], SBP <140 mmHg and LDL <2.5 mmol/L) and self-reported smoking cessation and daily physical activity – were evaluated using logistic regression, unadjusted and with the same adjustments as mentioned above. A composite outcome of reaching 3 versus 2 or fewer of the treatment goals was evaluated using logistic regression, with the same adjustments as above. Participants who died during follow-up were excluded from both the linear regression and the logistic regression analyses.

Cox regression analysis was used for the secondary outcome, all-cause death, unadjusted and adjusted as above. Other pre-specified subgroups were sex of dog owners, rural or urban area of living, number of dogs owned, income with subjects divided in four groups according to income quarters, and education divided into primary, secondary and tertiary education. Subgroup analyses of all-cause death were made with participants grouped according to smoking status, age quartiles, sex and self-reported daily physical activity. Cause of death was defined according to registered ICD-10 codes as: cardiovascular disease, I20 to I25 (ischemic heart disease), I45 to I52 (arrhythmic heart disease, heart failure and other heart diseases), I6 (cerebral infarction from occlusion or bleeding) or I70 to I74 (atherosclerosis, aortic aneurysm or dissection as well as arterial emboli or thrombi); cancer, C (malignant neoplasms) or D0 to D4 (*in situ* neoplasms, benign neoplasms and neoplasms of uncertain or unknown behaviour); diabetes mellitus, E10 to E14 (diabetes mellitus); and kidney failure, N17 to N19 (kidney failure).

An *ad hoc* matching analysis was made for dog owners vs. non-dog owners on a 1–2 ratio using propensity score matching for age, sex, hypertension medication, hyperlipidemia medication, systolic blood pressure, body weight, body mass index, LDL, physical activity, albuminuria, cholesterol, and smoking status. Matching was made with a caliper of 5 standard deviations for age. Baseline characteristics were evaluated for all the matched participants. Results were presented unadjusted and adjusted for variables which were not matched for: first HbA1c value, time to first recorded HbA1c from diabetes diagnosis, diabetes treatment, income quarter, education, and rural/urban area of living.

Statistical tests were two-tailed and *p*-values of <0.05 were considered statistically significant. R version 4.2.0 and RStudio version 2022.02.3 were used for data analyses.

## 3 Results

There were 279,635 patients diagnosed with type 2 diabetes between the years 2006–2016. After excluding participants with one or no registered HbA1c value or with follow up time less than 1 year between measurements, 218,345 were included in our analyses. Of these, 8,352 (3.8%) were dog owners, Table 1. There were 20,422 dogs registered amongst all the included participants. Median follow-up was 5.2 years (inter-quartile interval: 2.9 to 7.8) years.

**Table 1:** Baseline characteristics of dog owners and non-owners amongst newly diagnosed patients with type 2 diabetes in Sweden 2006–2016.

	Dog owners (*n* = 8,352)	Non-dog owners (*n* = 209,993)
Age at diabetes diagnosis, y – mean (SD)	55.2 (10.7)	62.6 (12.3)
Men – no. (%)	4,205 (50.3)	122,064 (58.1)
Systolic blood pressure, mmHg – mean (SD)	134.7 (15.9)	136.6 (16.8)
Diastolic blood pressure, mmHg – mean (SD)	81.0 (9.8)	79.5 (10.1)
HbA1c, mmol/mol – median (Q1–Q3)	50 (44–59)	49 (44–58)
HbA1c, % – median (Q1–Q3)	6.7 (6.2–7.5)	6.6 (6.2–7.5)
Cholesterol, mmol/L – median (Q1–Q3)	5.1 (4.4–6.0)	5.0 (4.3–5.8)
LDL, mmol/L – median (Q1–Q3)	3.0 (2.3–3.8)	2.9 (2.3–3.6)
HDL, mmol/L – median (Q1–Q3)	1.1 (1.0–1.4)	1.2 (1.0–1.4)
Triglycerides, mmol/L – median (Q1–Q3)	1.8 (1.2–2.6)	1.7 (1.2–2.4)
Creatinine, μmol/L – median (Q1–Q3)	69 (60–80)	73 (63–85)
eGFR, mL/min/1.73 m^2^ – median (Q1–Q3)	89.0 (76.6–102.3)	83.5 (70.0–99.3)
Moderately increased albuminuria – no. (%)	851 (10.2)	24,177 (11.5)
Severely increased albuminuria – no. (%)	249 (3.0)	7,884 (3.8)
Weight, kg – median (Q1–Q3)	93.0 (81.6–106.5)	88.0 (76.9–100.1)
Body mass index, kg/m^2^ – median (Q1–Q3)	31.5 (28.1–35.5)	29.9 (26.8–33.7)
Current cigarette smoking – no. (%)	1,658 (19.9)	32,252 (15.4)
Physically active on a daily basis – no. (%)	3,404 (40.8)	60,142 (28.6)
**Baseline treatment** – no. (%)		
Blood pressure lowering treatment	4,791 (57.4)	135,156 (64.4)
Diabetes treatment		
Lifestyle	3,411 (40.8)	95,733 (45.6)
Oral treatment	4,157 (49.8)	96,492 (46.0)
Insulin	323 (3.9)	8,203 (3.9)
Oral treatment and insulin	428 (5.1)	9,103 (4.3)
GLP-1 analogues in addition to oral treatment and/or insulin	27 (0.3)	295 (0.1)
Lipid-lowering treatment	3,199 (38.3)	88,205 (42.0)
**Socioeconomics**		
Education – no. (%)^a^		
Primary (year 1 to 9)	2,125 (25.4)	73,805 (35.1)
Secondary (year 10–11/12)	4,629 (55.4)	92,097 (43.9)
Tertiary (university)	1,545 (18.5)	38,216 (18.2)
Income quarters – no. (%)		
q1 (lowest)	2,043 (24.5)	51,626 (24.6)
q2	1,898 (22.7)	51,349 (24.5)
q3	2,178 (26.1)	52,048 (24.8)
q4	2,213 (26.5)	52,152 (24.8)
Area of living – no. (%)^b^		
Rural areas	5,188 (62.1)	103,490 (49.3)
Urban areas	3,144 (37.6)	103,685 (49.4)
**Number of dogs in the household** – no. (%)		
One	3,684 (44.1)	NA
Two	2,244 (26.9)	NA
Three or more	2,424 (29.0)	NA
**Number of dogs in the household – median (Q1–Q3)**	2 (1–3)	NA

Missing numbers (percentages) were 23,200 (10.6%) for triglycerides; 8,000–18,000 (4–8%) for cholesterol, HDL, LDL, albuminuria, and physically active on a daily basis; 2000–7,000 (1–3%) for eGFR, creatinine, body mass index, current cigarette smoking, and all socioeconomic variables; and less than 2000 (<1%) for all other variables. Differences between groups were tested using a chi square test for categorical variables and a one-way ANOVA and Mann–Whitney U test for continuous variables with approximately normal and skewed distribution, respectively. All comparisons between dog and non-dog owners had *p* < 0.001.

a Years 1 to 9 are mandatory for all Swedish school pupils. In the Swedish education system students can choose to study 2 or 3 years at a secondary level depending on orientation (most students study for 3 years). Admittance to university requires 3 years of secondary education.

b Classified by the Swedish Association of Local Authorities and Regions. Urban areas: large cities and medium-sized towns and municipalities near large towns or medium-sized towns. Rural areas: smaller towns/urban areas and rural municipalities. eGFR, estimated glomerular filtration rate; GLP-1, glucagon-like peptide-1; HDL, high-density lipoprotein; LDL, low-density lipoprotein; NA, not applicable.

Almost all participants, 216,998 (99.4%) had a registered first HbA1c within 2 years after diabetes diagnosis, with no difference between dog owners and non-dog owners, *p* = 0.320. Dog owners were younger at the diagnosis of diabetes than non-dog owners but had similar HbA1c and blood lipids. However, dog owners were more frequently smokers than non-dog owners (Table 1). Most dog owners, 5,928 (71.0%) had one or two dogs. Dog owners more often lived in rural areas compared to non-dog owners (*p* < 0.001). For dog owners, those with more than one dog were more likely to live in a rural area than the dog owners with one dog (3,034 [65.1%] vs. 2,154 [58.7%], *p* < 0.001).

Dog owners reported daily physical activity to a higher extent at end of follow-up compared to non-dog owners, OR 2.03 (1.93–2.13), *p* < 0.001, with 3,404 (42.3%) of dog-owners being daily physically active at baseline and 3,482 (43.2%) at end of follow-up. Non-dog owners reported being daily physically active to a lesser extent at end of follow-up (from 60,142 [29.9%] at baseline to 59,061 [29.3%] at end of follow-up).

**Figure 1:**
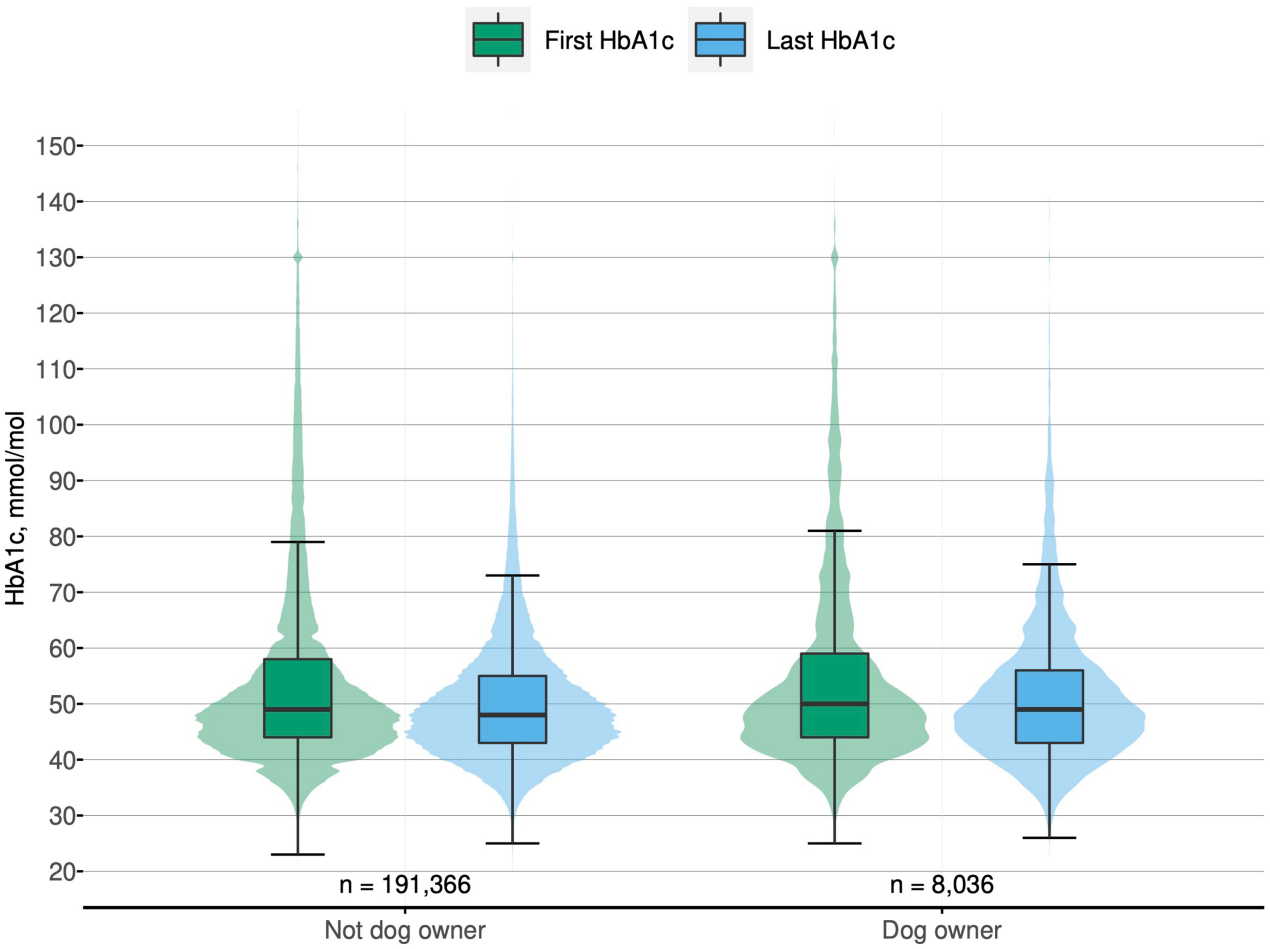
Combined box and violin plots showing the first and last HbA1c for non-dog and dog owners, respectively. The boxplot includes the median, the box extending between the 25th to the 75th percentile (the interquartile range, IQR) and its whiskers extending between the IQR times 1.5; the violin plot illustrates the relative distribution of observations. Participants that died during follow up were excluded from all analyses. To aid interpretation, outliers with HbA1c above 150 mmol/mol (15.9%) are not shown, *n* = 102 (0.05%). HbA1c, glycated haemoglobin.

Before and after adjustments, there was a difference between dog owners and non-dog owners for the primary outcome, yearly HbA1c change, where dog owners had a smaller mean change compared to non-dog owners; least square means −1.43 (−1.58 to −1.28) vs. −1.61 (−1.71 to −1.51) mmol/mol/year; *p* = 0.005, Table 2. Median HbA1c value decreased from the first to last value in both groups, Figure 1. After adjustments, dog owners were less likely than non-owners to reach the guideline recommended HbA1c goal of less than 52 mmol/mol (6.9%), LDL goal of less than 2.5 mmol/L and SBP goal of less than 140 mmHg adjusted ORs (95% CI): 0.93 (0.88–0.97), 0.91 (0.86–0.95) and 0.95 (0.90–1.00), respectively. Dog owners were less likely than non-dog owners to reach all 3 of these treatment goals vs. meeting 2 or fewer of them as well, (1,392 [17.3%] vs. 37,806 [19.8%]), OR 0.85 (0.80–0.90).

**Table 2:** Mean and 95% confidence interval (CI) for the main outcome change in HbA1c, odds ratio (95% CI) for guideline treatment goal achievement and lifestyle choices at follow up, and hazard ratio (95% CI) for all-cause mortality, for dog owners compared to non-dog owners with early type 2 diabetes.

	Unadjusted results		Adjusted for age and sex		Fully adjusted	
Primary outcome	Mean (95% CI)	*p*-value	Mean (95% CI)	*p*-value	Mean (95% CI)	*p*-value
Yearly change in HbA1c (mmol/mol/year)						
Dog owner	−1.39 (−1.54 to −1.23)	0.002	−1.39 (−1.55 to −1.22)	<0.001	−1.43 (−1.58 to −1.28)	0.005
Non dog owner	−1.64 (−1.67 to −1.60)		−1.64 (−1.69 to −1.58)		−1.61 (−1.71 to −1.51)	

Secondary outcomes	OR (95% CI)	*p*-value	OR (95% CI)	*p*-value	OR (95% CI)	*p*-value
Reaching guideline treatment goals						
HbA1c <52 mmol/mol (<6.9%)	0.83 (0.79 to 0.87)	<0.001	0.93 (0.89 to 0.97)	0.002	0.93 (0.88 to 0.97)	0.003
LDL <2.5 mmol/L	0.82 (0.78 to 0.86)	<0.001	0.92 (0.87 to 0.96)	<0.001	0.91 (0.86 to 0.95)	<0.001
SBP <140 mmHg	1.12 (1.07 to 1.17)	<0.001	0.92 (0.88 to 0.97)	0.001	0.95 (0.90 to 1.00)	0.045
Reaching all 3 vs. 2 or fewer of the above	0.85 (0.80 to 0.90)	<0.001	0.89 (0.84 to 0.94)	<0.001	0.90 (0.85 to 0.96)	0.001
Lifestyle choices at end of follow up						
Smokers that quit smoking during follow up^b^	0.97 (0.87 to 1.09)	0.626	1.01 (0.90 to 1.14)	0.872	1.00 (0.88 to 1.13)	0.977
Self-reported daily physical activity	1.77 (1.69 to 1.86)	<0.001	1.89 (1.80 to 1.98)	<0.001	2.03 (1.93 to 2.13)	<0.001

	HR (95% CI)	*p*-value	HR (95% CI)	*p*-value	HR (95% CI)	*p*-value
All-cause death^a^	0.41 (0.36 to 0.46)	<0.001	0.88 (0.78 to 0.98)	0.020	0.92 (0.81 to 1.04)	0.180

The primary outcome was analyzed using linear regression. The secondary outcome all-cause death was tested using Cox proportional hazards model. All other secondary outcomes were tested using generalized linear models. Adjustments were for sex, age, current smoking status, first HbA1c value, time to first recorded HbA1c from diabetes diagnosis, diabetes treatment, antihypertensive treatment, SBP, body mass index, LDL, lipid-lowering treatment, physical activity, income quartile, education, and rural/urban area of living. Secondary outcome models were not adjusted for the outcome measure. Participants that died during follow up were excluded from all analyses except for that of all-cause death. CI, confidence interval; HbA1c, glycated haemoglobin; HR, hazard ratio; LDL, low-density lipoprotein; OR, odds ratio; SBP, systolic blood pressure.

a During follow-up 316 (3.8%) of dog-owners and 18,627 (8.9%) of non-dog owners died.

b 33,910 of smokers at baseline reported smoking status during follow up. Out of these, 11,661 reported having quit. The models for smoking cessation were run in current smokers at baseline and not adjusted for smoking status.

**Figure 2:**
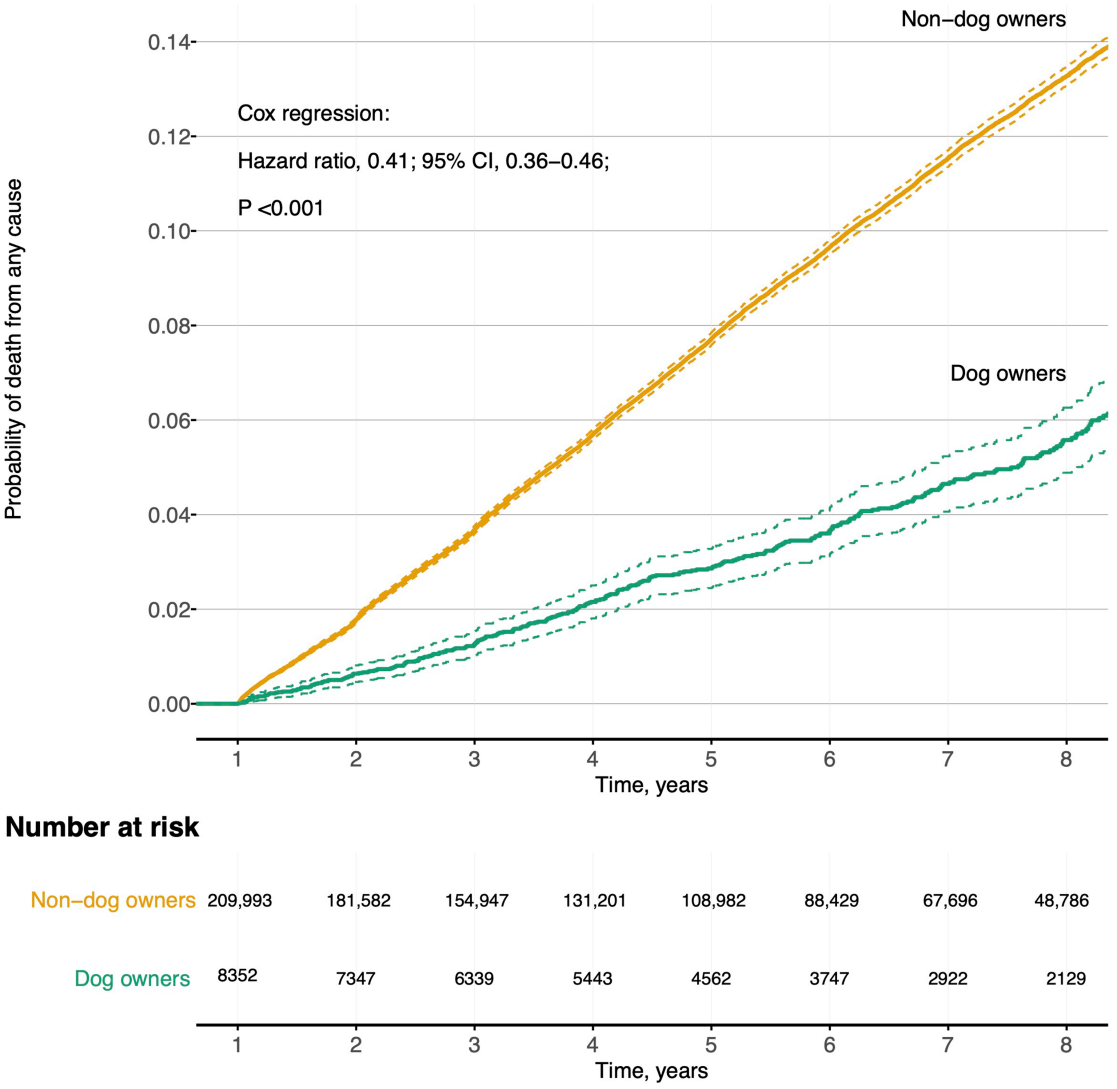
Cumulative incidence curve of unadjusted probability of death in dog owners and non-dog owners during follow-up. Participants with follow-up time beyond 8 years are not shown because of percentage at risk below 20%.

During follow-up, 18,943 (8.7%) participants died. The proportion of dog owners who died, 316 (3.8%) was lower than that amongst non-dog owners, 18,627 (8.9%); adjusted for age and sex, and fully adjusted, the HRs for all-cause death for dog owners compared to non-dog owners were 0.88 (0.78–0.98) and 0.92 (0.81–1.04), respectively, Table 2 and Figure 2. The most common causes of death for all participants were acute myocardial infarction (*n* = 1,599, 8.4%), chronic ischemic heart disease (*n* = 1,566, 8.3%) and lung cancer (*n* = 887, 4.7%); not shown. Amongst non-dog owners, CVD caused 31.9% (*n* = 5,942) of all deaths, whilst for dog-owners, CVD caused 21.8% (*n* = 69) of all deaths. However, after adjustments, there were no differences in cause of death between dog owners and non-dog owners; Supplementary Table S1. Dog owners in the oldest age quarter had a reduced risk of all-cause death, adjusted HR (95% CI), 0.76 (0.59–0.99); Table 3. But as dog owners were younger than non-dog owners there were fewer dog-owners in the oldest age quarter compared to non-dog owners (450 [5.4%] vs. 50,983 [24.3%]); not shown.

**Table 3:** Subgroup analyses of all-cause death, according to smoking status, age quartiles, sex and self-reported daily physical activity.

	Unadjusted results		Adjusted for age and sex*		Fully adjusted	
Secondary outcome, all-cause death	HR (95% CI)	*p*-value	HR (95% CI)	*p*-value	HR (95% CI)	*p*-value
Smoking status						
Smokers	0.54 (0.43 to 0.67)	<0.001	0.87 (0.70 to 1.09)	0.220	0.87 (0.68 to 1.11)	0.270
Non-smokers	0.38 (0.33 to 0.43)	<0.001	0.88 (0.77 to 1.00)	0.050	0.93 (0.80 to 1.07)	0.300
Age at diabetes diagnosis quartiles						
Q1	0.87 (0.67 to 1.14)	0.310	0.89 (0.68 to 1.17)	0.410	0.87 (0.64 to 1.17)	0.350
Q2	0.77 (0.63 to 0.95)	0.010	0.78 (0.64 to 0.97)	0.020	0.76 (0.60 to 0.96)	0.020
Q3	0.82 (0.67 to 1.01)	0.060	0.83 (0.67 to 1.01)	0.070	0.92 (0.73 to 1.15)	0.450
Q4	0.66 (0.52 to 0.82)	<0.001	0.63 (0.50 to 0.80)	<0.001	0.76 (0.59 to 0.99)	0.040
Sex						
Men	0.50 (0.43 to 0.57)	<0.001	0.87 (0.75 to 1.00)	0.050	0.90 (0.77 to 1.05)	0.190
Women	0.31 (0.26 to 0.37)	<0.001	0.90 (0.74 to 1.08)	0.250	0.96 (0.78 to 1.17)	0.670
Daily physical activity						
Yes	0.46 (0.38 to 0.55)	<0.001	0.89 (0.74 to 1.07)	0.200	0.90 (0.74 to 1.09)	0.270
No	0.41 (0.36 to 0.48)	<0.001	0.95 (0.82 to 1.10)	0.510	0.92 (0.79 to 1.08)	0.320

Models were adjusted for sex, age at diabetes diagnosis, current smoking status, first HbA1c value, time to first recorded HbA1c from diabetes diagnosis, diabetes treatment, antihypertensive treatment, systolic blood pressure, body mass index, low-density lipoprotein, lipid-lowering treatment, physical activity, income quartile, education, and rural/urban area of living. Models with population selection based on smoking status were not adjusted for current smoking status; models with population selection based on age were not adjusted for age; and models with population selection based on sex were not adjusted for sex. *Age quartiles were only adjusted for sex, and sex categories were only adjusted for age at diabetes diagnosis. CI, confidence interval; HbA1c, glycated haemoglobin; HR, hazard ratio.

In the *ad hoc* matching analysis, all results were comparable to the regression analyses, except that dog owners vs. non-dog owners no longer differed in terms of reaching the guideline recommended SBP goal of less than 140 mmHg, Supplementary Tables S2, S3.

## 4 Discussion

This study found that patients owning a dog when diagnosed with type 2 diabetes had worse glycaemic control than newly diagnosed type 2 diabetes patients that did not own a dog. Death during follow-up occurred less often amongst dog owners, but much of this difference could be attributed to confounding variables, principally age, leaving an 8% relative risk reduction in death associated with dog owning. However, dog owners were less likely to reach the guideline recommended goals for not only HbA1c but also LDL and SBP.

According to the official statistics from the dog register of the Swedish Board of Agriculture and Statistics Sweden, 335,000 (3.7%) and 631,000 (6.3%) of the adult Swedish population (aged 18 and above) were dog owners at December 31st 2006 and December 31st 2016, respectively (19, 20). In our study, the average proportion of patients with newly diagnosed type 2 diabetes that owned a dog, 3.8%, was similar to 2006 but far less than 2016, suggesting that over the study period, fewer patients with newly diagnosed diabetes owned a dog compared to the general public. This study was not designed to evaluate dog ownership as a preventive measure for diabetes, but the difference in age and smoking status in dog owners with type 2 diabetes compared to the non-dog owners with type 2 diabetes points towards the opposite and is interesting and worthy of further study.

Our findings regarding the comparative risk of death are consistent with those of a few recent studies, where dog ownership was found not to be associated with all-cause death (21, 22) or with CVD death (21). However, our results do not align with another Swedish study that found that dog ownership in the general population was associated with lower risk of all-cause death and cardiovascular death in both single- and multiple person households (7). A less sedentary behaviour and a healthier lifestyle in dog owners have previously been suggested as an explanatory factor for reduced mortality (7, 8, 13). In our study, dog owners reported being physically active to a higher extent at baseline as well as at end of follow up, and non-dog owners reported less likely to be active on daily basis which decreased during follow up. However, this did not impact on glucose control, treatment goal achievement or all-cause death, which is in line with a previous study (23).

Previously, some studies have shown that dog ownership was associated with both a lower prevalence of hypertension (24) and a greater risk of initiation of treatment for hypertension and dyslipidaemia in the general population (15). Thus, the collective evidence does not imply that owning a dog is strongly associated with health benefits. In addition, another study has found that owners of a dog with diabetes were themselves more likely to develop diabetes, which implied a shared diabetogenic health behaviour (25). Since we did not have any non-diabetic controls, we were not able to evaluate the lifestyle in healthy dog owners in comparison to those with type 2 diabetes. Furthermore, the mental wellbeing of dog ownership has previously been suggested as another factor involved in reduced mortality (7). Depression is twice as common in patients with diabetes compared to people without diabetes (26) and comorbid depression in patients with type 2 diabetes increases the risk of early onset, and progression of micro- and macrovascular disease and all-cause death (14). Symptoms of depression can be reduced and health related quality of life can be improved by physical activity and physical fitness in patients with type 2 diabetes (27). Owning a pet has been shown to increase psychological wellbeing (28) and this has been shown for dogs specifically (13).

### 4.1 Strengths and limitations

The strength of this study is that the four national registers used have overall excellent coverage. It is mandatory to register all domestic dogs in Sweden. Statistics Sweden has full coverage due to the Swedish personal identity number system. NDR has a coverage of approximately 87%, based on comparison of registered patients with type 2 diabetes in NDR to patients collecting medications for diabetes (29). Limitations are the lack of important explanatory variables, such as medical history or concomitant disease. In our study we had no means of evaluating mental health, and thus cannot adjust for, or consider current depressive symptoms or quality of life in the cohort, but this potential mental wellbeing did not translate to any hard outcomes such as glucose control or mortality in our study. Physical activity was self-reported, rather than measured. Other limitations are that whether the associations seen for dog owners are due to the ownership itself, or factors underlying the decision to get a dog are not possible to elaborate on. Also, dog ownership is registered per household and not per individual, which means that for shared households, we cannot determine whether the individual studied is the actual caretaker of the dog. Furthermore, our study did not include healthy individuals with or without dogs for comparison. Finally, we did not analyse the breed or size of the dogs owned, as previous studies have not shown significant differences between dog breeds and physical activity (30).

## 5 Conclusion

To the best of our knowledge, this is the first study to evaluate the association between dog ownership and glucose control and mortality in patients with newly diagnosed type 2 diabetes. Owning a dog at diabetes diagnosis, or getting one within the first year after diagnosis, did not improve glucose control, treatment goal achievement for blood pressure, LDL or reduced the risk for all-cause death, although dog-owners were more daily physically active than non-dog owners.

## Data availability statement

The data analyzed in this study is subject to the following licenses/restrictions: the data that support the findings of this study are available on request from the corresponding author. The data are not publicly available due to privacy or ethical restrictions. Requests to access these datasets should be directed to olov.rolandsson@umu.se.

## Ethics statement

The studies involving humans were approved by the Regional Ethical Review Board in Umeå. The studies were conducted in accordance with the local legislation and institutional requirements. Written informed consent for participation was not required from the participants or the participants’ legal guardians/next of kin in accordance with the national legislation and institutional requirements.

## Author contributions

KR: Writing – original draft. PaG: Writing – review & editing. MW: Writing – review & editing. JC: Writing – review & editing. MH: Writing – review & editing. SJ: Writing – review & editing. OR: Writing – review & editing.
