# Supplementary material for: Dog ownership, glycaemic control and all-cause death in patients with newly diagnosed type 2 diabetes: a national cohort study
Source: Front Public Health. 2023 Dec 15;11:1265645. doi: 10.3389/fpubh.2023.1265645 (PMC10757647; doi:10.3389/fpubh.2023.1265645)
Supplement: Supplementary file 1 [file Data_Sheet_1.docx]

**Supplementary table 1.** Odds ratios and 95% CI for cause of death for dog owners compared with non-dog owners according to age quarters. Presented for cardiovascular disease (CVD) versus all other causes and cancer versus all other causes respectively.

|  | | **Age at diabetes diagnosis, quarters** | | | | | | | | **All patients,**  N = 18,943 | |
| --- | --- | --- | --- | --- | --- | --- | --- | --- | --- | --- | --- |
|  |  | **1^st^ (21 – 54 y),**  n = 924 | | **2^nd^ (54 – 63 y),**  n = 2543 | | **3^rd^ (63 – 72 y),**  n = 4308 | | **4^th^ (72 – 103 y),**  n = 11,168 | |  |  |
|  |  | OR (95% CI) | p-value | OR (95% CI) | p-value | OR (95% CI) | p-value | OR (95% CI) | p-value | OR (95% CI) | p-value |
| **CVD** | **Unadjusted** | 0.55 (0.24-1.30) | 0.174 | 0.97 (0.54-1.73) | 0.911 | 0.66 (0.38-1.15) | 0.142 | 0.93 (0.53-1.64) | 0.798 | 0.70 (0.52-0.94) | 0.018 |
|  | **Adjusted** | 0.56 (0.20-1.56) | 0.265 | 0.89 (0.46-1.72) | 0.736 | 0.64 (0.35-1.19) | 0.157 | 0.96 (0.51-1.84) | 0.909 | 0.82 (0.58-1.15) | 0.245 |
| **Cancer** | **Unadjusted** | 1.32 (0.74-2.35) | 0.341 | 1.27 (0.78-2.05) | 0.334 | 0.86 (0.54-1.37) | 0.528 | 1.54 (0.89-2.67) | 0.124 | 1.37 (1.07-1.77) | 0.014 |
|  | **Adjusted** | 1.03 (0.51-2.09) | 0.924 | 1.17 (0.68-2.02) | 0.566 | 0.82 (0.49-1.37) | 0.445 | 1.35 (0.72-2.53) | 0.342 | 1.12 (0.85-1.50) | 0.419 |

Linear regression of participants that died during follow-up. The model was adjusted for sex, age at diabetes diagnosis, current smoking status, first glycated haemoglobin value, time to first recorded HbA1c from diabetes diagnosis, diabetes treatment, antihypertensive treatment, systolic blood pressure, body mass index, low-density lipoprotein, lipid-lowering treatment, physical activity, income quartile, education, and rural/urban area of living. CVD, cardiovascular disease.

**Supplementary table 2.** Baseline characteristics of dog owners and non-owners among newly diagnosed patients with type 2 diabetes in Sweden 2006-2016 after matching.

|  | **Dog owners**  **(n = 6928)** | **Non-dog owners**  **(n = 13,850)** |
| --- | --- | --- |
| Age at diabetes diagnosis, y – mean (SD) | 55.2 (10.6) | 55.2 (10.5) |
| Men – no. (%) | 3514 (50.7) | 7196 (52.0) |
| Systolic blood pressure, mmHg – mean (SD) | 134.8 (15.9) | 135.2 (16.4) |
| Diastolic blood pressure, mmHg – mean (SD) | 81.0 (9.8) | 81.1 (10.1) |
| HbA1c, mmol/mol – median (Q1-Q3) | 50 (44-59) | 50 (44-60) |
| HbA1c, % – median (Q1-Q3) | 6.7 (6.2-7.5) | 6.7 (6.2-7.6) |
| Cholesterol, mmol/L – median (Q1-Q3) | 5.2 (4.4-6.0) | 5.2 (4.5-5.9) |
| LDL, mmol/L – median (Q1-Q3) | 3.0 (2.3-3.8) | 3.1 (2.5-3.7) |
| HDL, mmol/L – median (Q1-Q3) | 1.1 (1.0-1.4) | 1.1 (1.0-1.4) |
| Triglycerides, mmol/L – median (Q1-Q3) | 1.8 (1.2-2.6) | 1.8 (1.3-2.5) |
| Creatinine, μmol/L – median (Q1-Q3) | 69 (60-80) | 69 (60-80) |
| eGFR, mL/min/1.73 m^2^ – median (Q1-Q3) | 88.9 (76.7-102.2) | 89.2 (76.6-103.6) |
| Moderately increased albuminuria – no. (%) | 753 (10.9) | 1472 (10.6) |
| Severely increased albuminuria – no. (%) | 214 (3.1) | 434 (3.1) |
| Weight, kg – median (Q1-Q3) | 93.0 (81.7-106.0) | 92.9 (81.0-106.2) |
| Body mass index, kg/m^2^ – median (Q1-Q3) | 31.4 (28.0-35.4) | 31.3 (28.1-35.4) |
| Current cigarette smoking – no. (%) | 1367 (19.7) | 2827 (20.4) |
| Physically active on a daily basis – no. (%) | 2976 (43.0) | 5979 (43.2) |
| **Baseline treatment** – no. (%) |  |  |
| Blood pressure lowering treatment | 3951 (57.0) | 8080 (58.3) |
| Diabetes treatment |  |  |
| Lifestyle | 2870 (41.4) | 5783 (41.8) |
| Oral treatment | 3421 (49.4) | 6911 (49.9) |
| Insulin | 259 (3.7) | 449 (3.2) |
| Oral treatment and insulin | 358 (5.2) | 680 (4.9) |
| GLP-1 analogues in addition to oral treatment and/or insulin | 20 (0.3) | 23 (0.2) |
| Lipid-lowering treatment | 2652 (38.3) | 5367 (38.8) |
| **Socioeconomics** |  |  |
| Education – no. (%)^§^ |  |  |
| Primary (year 1 to 9) | 1758 (25.4) | 3785 (27.3) |
| Secondary (year 10-11/12) | 3836 (55.4) | 6777 (48.9) |
| Tertiary (university) | 1290 (18.6) | 2891 (20.9) |
| Income quarters – no. (%) |  |  |
| q1 (lowest) | 1659 (23.9) | 3627 (26.2) |
| q2 | 1566 (22.6) | 3071 (22.2) |
| q3 | 1831 (26.4) | 3465 (25.0) |
| q4 | 1855 (26.8) | 3464 (25.0) |
| Area of living – no. (%)^¥^ |  |  |
| Rural areas | 4314 (62.3) | 6646 (48.0) |
| Urban areas | 2597 (37.5) | 6981 (50.4) |
| **Number of dogs in the household** – no. (%) |  |  |
| One | 3077 (44.4) | NA |
| Two | 1881 (27.2) | NA |
| Three or more | 1970 (28.4) | NA |
| **Number of dogs in the household – median (Q1-Q3)** | 2 (1-3) | NA |

Matching was made using propensity score matching on a 1 – 2 ratio for age (using a caliper of 5 standard deviations), sex, hypertension medication, hyperlipidemia medication, systolic blood pressure, body weight, body mass index, low-density lipoprotein, physical activity, albuminuria, cholesterol, and smoking status.

Differences between groups were tested using a chi square test for categorical variables and a one-way ANOVA and Mann-Whitney U test for continuous variables with approximately normal and skewed distribution, respectively. Comparisons between dog and non-dog owners with *P* <.001 were education, income quarters, and area of living.

**Supplementary table 3.** Results after 1 – 2 ratio propensity score matching. Mean and 95% confidence interval (CI) for the main outcome change in HbA1c, odds ratio (95% CI) for guideline treatment goal achievement and lifestyle choices at follow up, and hazard ratio (95% CI) for all-cause mortality, for dog owners compared to non-dog owners with early type 2 diabetes.

|  | **Unadjusted** | | **Fully adjusted** | |
| --- | --- | --- | --- | --- |
| **Primary outcome** | **Mean (95% CI)** | **p-value** | **Mean (95% CI)** | **p-value** |
| **Yearly change in HbA1c (mmol/mol/year)** |  |  |  |  |
| Dog owner | -1.40 (-1.57 to -1.23) | <0.001 | -1.60 (-1.73 to -1.47) | 0.002 |
| Non dog owner | -1.93 (-2.05 to -1.80) |  | -1.84 (-1.93 to -1.75) |  |
|  |  |  |  |  |
| **Secondary outcomes** | **OR (95% CI)** | **p-value** | **OR (95% CI)** | **p-value** |
| **Reaching guideline treatment goals** |  |  |  |  |
| HbA1c <52 mmol/mol (<6.9 %) | 0.91 (0.86 to 0.97) | 0.003 | 0.92 (0.86 to 0.98) | 0.012 |
| LDL <2.5 mmol/L | 0.92 (0.86 to 0.97) | 0.004 | 0.93 (0.88 to 0.99) | 0.027 |
| SBP <140 mmHg | 0.98 (0.92 to 1.04) | 0.534 | 1.00 (0.94 to 1.06) | 0.944 |
| Reaching all 3 vs 2 or fewer of the above | 0.90 (0.83 to 0.97) | 0.005 | 0.92 (0.85 to 0.99) | 0.030 |
|  |  |  |  |  |
| **Lifestyle choices at end of follow up** |  |  |  |  |
| Smokers that quit smoking during follow up | 1.01 (0.87 to 1.17) | 0.947 | 1.04 (0.89 to 1.21) | 0.599 |
| Self-reported daily physical activity | 1.62 (1.52 to 1.72) | <0.001 | 1.63 (1.54 to 1.74) | <0.001 |
|  | **HR (95% CI)** | **p-value** | **HR (95% CI)** | **p-value** |
| **All-cause death** | 0.91 (0.78 to 1.06) | 0.219 | 0.91 (0.78 to 1.07) | 0.251 |

Matching was made using propensity score matching on a 1 – 2 ratio for age (using a caliper of 5 standard deviations), sex, hypertension medication, hyperlipidemia medication, systolic blood pressure, body weight, body mass index, low-density lipoprotein, physical activity, albuminuria, cholesterol, and smoking status. The primary outcome was analysed using linear regression. The secondary outcome all-cause death was tested using Cox proportional hazards model. All other secondary outcomes were tested using generalized linear models. Participants that died during follow up were excluded from all analyses except for that of all-cause death. Adjustments were made for variables which were not matched for: first HbA1c value, time to first recorded HbA1c from diabetes diagnosis, diabetes treatment, income quarter, education, and rural/urban area of living.

CI = confidence interval, HbA1c = glycated haemoglobin, HR = hazard ratio, LDL = low-density lipoprotein, OR = odds ratio, SBP = systolic blood pressure.
